# Antimicrobials from Seaweeds for Food Applications

**DOI:** 10.3390/md19040211

**Published:** 2021-04-11

**Authors:** Eduarda M. Cabral, Márcia Oliveira, Julie R. M. Mondala, James Curtin, Brijesh K. Tiwari, Marco Garcia-Vaquero

**Affiliations:** 1 Teagasc, Food Research Centre, Ashtown, 15 Dublin, Ireland; eduarda.neves@teagasc.ie (E.M.C.); brijesh.tiwari@teagasc.ie (B.K.T.); 2 Department of Food Hygiene and Technology and Institute of Food Science and Technology, University of León, 24071 León, Spain; msouo@unileon.es; 3 School of Food Science & Environmental Health, College of Sciences & Health, Technological University Dublin-City Campus, 7 Dublin, Ireland; julie.mondala@tudublin.ie (J.R.M.M.); james.curtin@tudublin.ie (J.C.); 4 School of Agriculture and Food Science, University College Dublin, Belfield, 4 Dublin, Ireland

**Keywords:** antimicrobial bioactives, novel compounds, seaweed, foodborne pathogens, shelf-life, preservation, sustainability

## Abstract

The exponential growth of emerging multidrug-resistant microorganisms, including foodborne pathogens affecting the shelf-life and quality of foods, has recently increased the needs of the food industry to search for novel, natural and eco-friendly antimicrobial agents. Macroalgae are a bio-diverse group distributed worldwide, known to produce multiple compounds of diverse chemical nature, different to those produced by terrestrial plants. These novel compounds have shown promising health benefits when incorporated into foods, including antimicrobial properties. This review aims to provide an overview of the general methods and novel compounds with antimicrobial properties recently isolated and characterized from macroalgae, emphasizing the molecular pathways of their antimicrobial mechanisms of action. The current scientific evidence on the use of macroalgae or macroalgal extracts to increase the shelf-life of foods and prevent the development of foodborne pathogens in real food products and their influence on the sensory attributes of multiple foods (i.e., meat, dairy, beverages, fish and bakery products) will also be discussed, together with the main challenges and future trends of the use of marine natural products as antimicrobials.

## 1. Introduction

Over the past few years, food industries have been challenged to increase and improve food production in a sustainable and competitive manner, leading to the increased implementation of green technologies [1] and the use of natural molecules in food formulations, replacing traditionally used ingredients and preservatives [2]. These novel strategies must agree with the existing food safety regulations, while assuring and providing high quality and nutritious food. To maintain and improve their competitiveness and high standards, food industries are looking at innovative alternatives to produce healthy, safe, convenient and natural foodstuffs with extended shelf-life [2].

The imminent need to define and develop novel, natural and eco-friendly antimicrobial agents has greatly increased in the past few years due to the exponential growth of emerging multidrug-resistant microorganisms, such as *Listeria monocytogenes*, *Staphylococcus aureus*, *Escherichia coli*, *Salmonella* spp., *Vibrio parahaemolyticus*, *Pseudomonas aeruginosa* and *Enterobacter* spp., amongst others [3,4]. Natural antimicrobial agents currently investigated for this purpose include multiple compounds of plant origin, such as essential oils and phenolic compounds [2], acids from natural fermentation processes (vinegars), animal/microbial origin compounds (i.e., peptides, lipids, chitosan, lactoperoxidase systems, bacteriocins and lysozymes) and the use of competitive microbiota and bacteriophages [5,6,7]. The use of these novel compounds in food matrices must possess ideally equal or higher efficiency when extending the shelf-life of foods compared to traditional preservatives, while maintaining and/or improving the characteristic sensory attributes of the food products that may influence consumers’ perception and acceptability.

Within the market of natural products, seaweeds or macroalgae have gained momentum as a green and sustainable biomass for human and animal consumption. In Asian countries, the consumption of seaweeds, such as *Laminaria digitata*, *Ascophyllum nodosum*, *Fucus vesiculosus*, *Macrocystis pyrifera*, *Sargassum fusiforme*, *Undaria pinnatifida*, *Porphyra* spp. (nori) and *Monostroma* spp. (aonori), dates back to ancient times [8,9], and the incorporation of seaweeds into the diet in western countries is currently increasing, being included in a wide variety of foodstuffs, such as soups and salads [10]. However, only in recent years, seaweeds have attracted the attention of the research community as a new source of nutritional ingredients, different in composition to those of terrestrial plants, and bioactive compounds with multiple health benefits including antimicrobial properties [11,12,13,14]. Macroalgae have evolved to survive multiple threats in the marine environment, including high concentrations of infectious and surface-fouling bacteria that are indigenous to oceans, by developing distinct mechanisms of defense, such as the production of antimicrobial compounds [15,16,17,18]. Moreover, the macroalgal biomass encompasses a wide variety of species—classified as Chlorophyta (green algae), Rhodophyta (red algae) and Phaeophyceae (brown algae)—widely distributed all over the world; thus, the exploration of this diverse biomass as a source of antimicrobials for food and non-food applications appears to be an unlimited field of study.

This review aims to provide an overview of the methods used to test the antimicrobial potential of novel natural compounds together with the recent advances in the discovery of novel antimicrobials from macroalgae and their mechanisms of action. The current scientific evidence on the use of macroalgae or macroalgal extracts to increase the shelf-life of foods and prevent the development of foodborne pathogens in real food products and their influence on the sensory attributes of multiple foods (i.e., meat, dairy, beverages, fish and bakery products) will also be covered, together with the main challenges and future trends of the use of natural products from seaweeds as antimicrobials.

## 2. Methods to Test the Antimicrobial Properties of Novel Compounds

Several methods are widely used by researchers to identify and quantify the antimicrobial activity of natural compounds from multiple sources, including macroalgae. Due to the wide variety of tests to assess the antimicrobial properties of compounds, the comparison of these activities between multiple studies is difficult to interpret. In the current scientific literature, most authors assess natural compounds following in vitro susceptibility methods or in vivo tests [11].

### 2.1. Antimicrobial Susceptibility Methods

Antimicrobial susceptibility testing methods (AST) are in vitro techniques based on phenotypic tests to determine the efficacy of potential antimicrobials at defined concentrations against a given microorganism. These methods are considered cheap and easy to implement as well as having a straightforward interpretation. AST methods are growth-based methods that compare visible microbial growth at specific antimicrobial concentrations, providing a quantitative assessment of the minimal inhibitory concentration (MIC). The MIC value is the lowest concentration of an antimicrobial compound that prevents the visible growth of any microorganism [19].

On the other hand, antimicrobial resistance (AMR) detection methods based on genomics, transcriptomics and proteomics tools are used to detect or predict antimicrobial resistance. Molecular AMR methods are used not to directly measure the cell viability but to detect changes and/or expression on specific resistance genes, as well as mutations [20]. These methods are well-established, allowing researchers to obtain rapid results in extensive applications. However, the costs and the insufficient availability of user-friendly bioinformatics programs are important barriers for the implementation of the molecular approaches as a routine application.

Several AST methods are widely used in research to detect and measure the efficacy of macroalgal bioactives and the selection of the method of choice will depend on the intended degree of accuracy, convenience, as well as availability of resources and technical expertise. The most commonly used methods to determine MIC values include the disc diffusion method and dilution method (broth and agar dilution method) [21].

#### 2.1.1. Disc Diffusion Method

The diffusion methods are recommended by the National Committee for Clinical Laboratory Standards (NCCLS) and they have been used to screen the antimicrobial activity of multiple macroalgal extracts [22,23,24]. In the agar diffusion method, an agar plate is first evenly seeded with the microbial isolate of interest, and then filter paper discs, wells or strips impregnated with a known concentration of the compound to be tested are placed on it [25]. The tested compound will diffuse onto the agar and the antimicrobial activity is measured as the growth inhibition zone around the disc. The size of the inhibition zone and the corresponding compound concentration will determine the MIC values [25].

#### 2.1.2. Dilution Method

Analysis of antimicrobial activity using dilution methods is based either on the limited growth or no visible growth on solid agar plate, or on the lack of visible turbidity in broth medium after an appropriate incubation. The broth (micro) dilution method has been used in several studies using algal extracts [26,27,28]. This method, also called the microtiter plate method, is widely used, mainly for a large number of test samples, and provides robust and useful results of MIC values. The broth dilution method has been standardized by the NCCLS and the European Committee on Antimicrobial Susceptibility Testing (EUCAST) [29]. The method consists in using a liquid growth medium inoculated with the tested microorganism, to which a series of increasing and known concentrations of the antimicrobial compound are added. The lowest concentration in which there is no evidence of visible cloudiness or sedimentation after a period of incubation is registered as the MIC [21]. Another way to measure the growth or no growth of an isolate is using a 96-well plate, where the turbidity can be measured by recording the optical density (OD) using a plate reader [30]. In addition, the membrane damage caused by the antimicrobial compound can also be measured using dyes such as SybrGreen^®^ [31]. These dyes penetrate the cell, generating a change in their fluorescence [32].

Another technique similar to broth dilution is agar dilution. In the agar dilution method, the tested microorganisms are spot-inoculated onto the agar medium containing serially diluted antimicrobial concentrations. This method is useful to establish the lowest concentration of the antimicrobial at which bacterial growth is still inhibited.

#### 2.1.3. Molecular AMR Methods

With the emergence and spread of antimicrobial resistance worldwide, it is of great importance to develop and apply new methods to identify the presence of genes or genetic variations responsible for antibiotic resistance. Several genotypic methods have been used for the rapid detection of AMR genes, including nucleic acid amplification methods, particularly real-time quantitative PCR (qPCR), DNA hybridization-based methods, mainly DNA microarrays, Luminex xMAP technology and next-generation sequencing methods [33,34,35]. However, most of these molecular detection approaches only target well-studied microorganisms or resistance genes and do not easily enable the detection of novel genes. The recent development of “omics” approaches has paved the way to the identification of novel resistance genes and provided access to genetic diversity [36]. Whole-genome sequencing (WGS) or next-generation sequencing (NGS) allow the assessment of genomes, delivering high-resolution genotyping results in a short period of time [37]. The application of WGS/NGS allows the prediction of antimicrobial resistance/susceptibility [38] and could be a useful tool to be used in routine diagnosis, replacing traditional cultural approaches in the near future.

### 2.2. In Vivo Assays

Current in vitro AST studies are typically limited to obtaining the antimicrobial capacity of compounds when used in controlled environments. These techniques are performed under laboratory conditions that are not generally met when using in vivo models. Therefore, after the assessment of the antimicrobial properties in vitro of macroalgal compounds, it is beneficial to study the antimicrobial efficacy, as well as the toxicity, of the compounds in vivo using relevant experimental models.

Several in vivo models have been used in these types of studies, and, in general, the most widely used include zebrafish, mice and rats [4,39,40,41,42]. However, in vivo tests are expensive, can take a long time to deliver accurate results and involve ethical issues regarding animal testing. To date, most in vivo testing of macroalgal extracts has used non-vertebrates or cold-blooded vertebrates [39].

Little information exists in the literature regarding the evaluation of antimicrobial activity from marine algae compounds using in vivo assays. Some of the in vivo assays used farmed fish and shrimp as animal models against various pathogens [43]. Additionally, Vatsos and Rebours [43] reported that in aquaculture studies, the macroalgal compounds were either incorporated directly in the feeds (dry or live) or added directly into the water in which the fish and shrimp were grown. The efficacy of microalgae such as *Chlorella vulgaris* or *Arthrospira platensis* [44,45] and the polysaccharide fraction of the *Caulerpa scalpelliformis* or *Padina gymnospora* [46,47] was reported in Nile tilapia (*Oreochromis niloticus*) and common carp (*Cyprinus carpio*). In addition, microencapsulated macroalgal extracts were tested against *Aeromonas salmonicida* in *Oreochromis mossambicus* [48]. Additionally, Manilal et al. [49] studied the therapeutic potential of algal extracts on shrimp by oral administration, and other researchers performed assays to demonstrate the antibacterial protection on fish by immersion application [48,50].

## 3. Macroalgal Compounds with Antimicrobial Properties and Their Mechanisms of Action

Macroalgae reside in a widely varied taxonomic group that encompasses over 10,000 species surviving the extreme environmental conditions of the sea by producing unique secondary metabolites including phlorotannins, proteins and polysaccharides, different in composition to those found in terrestrial plants [14]. One of the functions of these secondary metabolites, amongst others described in the literature (i.e., antioxidants), is their role as defense mechanisms against pathogens, fouling organisms and herbivores in seaweeds. Thus, this biodiverse group offer excellent opportunities for the discovery of novel, biologically active, natural compounds with higher efficacy against microorganisms compared to known antimicrobials.

The active chemical compounds responsible for the antibacterial properties in algae have been identified as phenols, fatty acids, carbohydrates, proteins and other minor compounds [51,52,53,54]. The chemical structure and relative abundance of these molecules in macroalgae is extremely variable depending on environmental factors that vary during the year (i.e., salinity, pH, solar irradiation, temperature and nutrients), the reproductive stage of the biomass and parts of the macroalgae sampled [55], together with other stressors affecting the biomass, such as competition with other plants and the presence of animal grazers and pathogens [56]. Due to the varied chemical nature and the complexity of these molecules, the majority of studies available on the antimicrobial potential of macroalgae focus on assessing the in vitro antimicrobial properties of various macroalgal extracts obtained using multiple solvents. These extracts contain multiple and complex mixtures of compounds that, in the majority of the cases, are not chemically characterized, as reviewed by Pérez et al. [57]. The preliminary evaluation of these crude extracts from macroalgae should be followed by more complex purification procedures, such as bioactive-guided fractionation processes, to allow the characterization of the chemical species responsible for the antimicrobial properties and their antimicrobial mechanisms of action for their future industrial exploitation as antimicrobials. The main antimicrobial compounds recently isolated and characterized from multiple seaweeds and the main antimicrobial properties of these compounds are compiled in Table 1.

### 3.1. Phenolic Compounds

Phenolic compounds or phenols are a class of molecules consisting of one or more hydroxyl groups directly attached to an aromatic hydrocarbon group [80]. Algal polyphenols derive from polymerized phloroglucinol units (1,3,5-trihydroxybenzene) and their variety is dependent upon on the number of phenolic rings and the radicals bound to those rings [81]. Within the phenolics group with proven health beneficial properties, phlorotannins and bromophenols have recently gained attention as potential antimicrobials, as seen by the wide variety of compounds recently characterized from macroalgae in recent research (see Table 1).

#### 3.1.1. Phlorotannins

**Figure 1:**
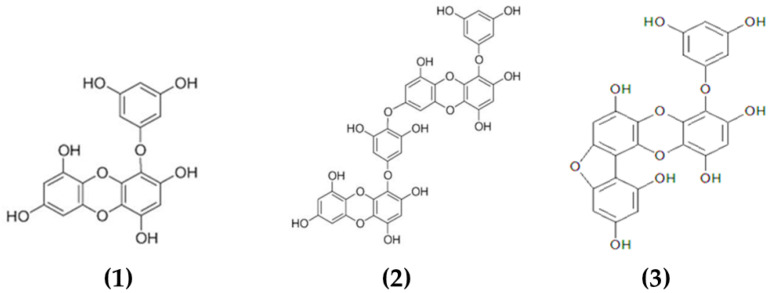
Chemical structures of various phlorotannins isolated from macroalgae. The phlorotannins in the image are: (**1**) eckol, (**2**) dieckol, (**3**) fucofuroeckol-A.

Phlorotannins constitute a heterogeneous group of molecules, with molecular sizes ranging from 0.126 to 650 kDa, constituted by polymerized phloroglucinol (1,3,5-trihydroxybenzene) monomer units and biosynthesized through the acetate–malonate pathway or polyketide pathway [82]. Brown macroalgae can accumulate high amounts of phlorotannins, representing between 5 and 15% of the dried weight of the biomass depending on the species and season of collection [83]. Based on their molecular weight, these compounds can be characterized as low-, intermediate- and high-molecular-weight phlorotannins containing phenyl and phenoxy units. The type of linkage will further serve to classify these compounds into four subclasses, namely fuhalols and phlorethols (ether linkage), fucols (phenyl linkage), fucophloroethols (ether and phenyl linkage) and eckols (dibenzodioxin linkage) [82]. The complexity of the chemical structures of characterized phlorotannins can be seen in Figure 1.

Multiple biological properties and health benefits have been attributed to macroalgal phlorotannins, including antioxidant, anti-allergic, anti-inflammatory and antimicrobial [84,85,86,87]. However, limited information is available in the literature on the chemical characterization of these compounds. Predominantly, studies focused on the characterization of low-molecular-weight phlorotannins (2–8 monomeric units) that represent a relatively small proportion of all the phlorotannins available in macroalgae. Several phlorotannins isolated and characterized from macroalgae and with demonstrated antimicrobial properties include dieckol [58], eckol [59] and fucofuroeckol-A [60] (see Table 1).

Choi et al. [59] determined the promising antibacterial properties of eckol isolated from *Ecklonia cava* against *S. aureus* and *Salmonella* strains, with MICs ranging from 125 to 250 μg/mL. Moreover, the authors also appreciated that combinations of eckol and a known antibiotic (ampicillin) improved the inhibition of *S. aureus* and *Salmonella* strains due to a synergic or additive effect [59]. Other authors also appreciated the positive effects of phlorotannins on reducing the antibacterial resistance in combined treatments with antibiotics. Lee et al. [58] reported that dieckol from *Ecklonia cava* was able to reverse the high resistance to ampicillin and penicillin of MRSA, reducing the MICs of ampicillin against two strains of MRSA from 512 to 0.5 μg/mL in combination with 1/4 MIC of dieckol (16 μg/mL). Moreover, the authors suggested that combinations of dieckol–ampicillin or dieckol–penicillin had a synergistic effect against MRSA and, thus, these combinations may be used for the development of alternative phytotherapeutic treatments against MRSA bacteria [58]. Other phlorotannins such as fucofuroeckol-A from *Eisenia bicyclis*, exhibited the highest antibacterial activity against acne-related bacteria (MIC 32–128 μg/mL) and had also a weak synergistic effect with erythromycin and lincomycin that could have potential when developing future acne treatments using natural products [60].

The mechanisms of action of the antimicrobial effect of phlorotannins have not been clearly elucidated. Previous reports suggest that the antibacterial activity exerted by some phlorotannins may be related to an inhibition of the oxidative phosphorylation pathway in the microorganisms and the capacity of these molecules to cohere with microbial proteins, such as cell membranes and enzymes, causing cell disintegration [88,89,90]. Hierholtzer et al. [88] appreciated that cells exposed to phloroglucinol from *Laminaria digitata* appeared as spore-like structures, with disrupted outer membranes, coagulation of exo-polysaccharides, separation of the cytoplasmic membrane from the cell envelope and “blebbing” and debris of components coagulated by phlorotannins. The microscopic observations of cells exposed to phloroglucinol suggested that this compound interacts with the bacterial envelope and triggers survival mechanisms in the microbes. These authors concluded that the bactericidal activity of phlorotannins seems related to the level of polymerization of the compounds, as the disturbance of the cells’ envelopes is the key step associated with the bactericidal action of phlorotannins [88]. Moreover, Eom et al. [91] also reported the antibacterial effect of phlorofucofuroeckol-A on MRSA KCCM 40511. The cells were disrupted and the intracellular material extruded in a dose- and time-dependent manner following exposure to phlorofucofuroeckol-A [91]. Furthermore, low-molecular-weight phlorotannins from *Sargassum thunbergii* also showed similar effects against *V. parahaemolyticus* [92]. These compounds inhibited the growth of the bacteria during the logarithmic phase and damaged their cell walls and membranes, leading to the leakage of cytoplasm and deconstruction of membrane permeability [92].

#### 3.1.2. Bromophenols and Other Halogenated Compounds

**Figure 2:**
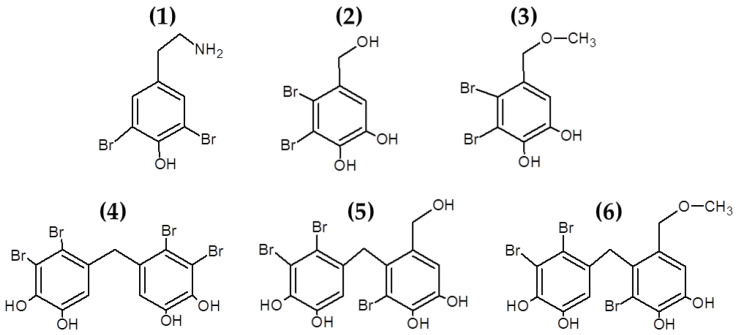
Chemical structure of bromophenols from *Odonthalia corymbifera*. The compounds in the figure correspond to: (**1**) (2-aminoethyl)-2,6-dibromophenol; (**2**) 19, 20 2,3-dibromo-4,5-dihydroxybenzyl alcohol; (**3**) 10 2,3-dibromo-4,5-dihydroxybenzyl methyl ether; (**4**) 17 2,2′,3,3′-tetrabromo-4,4′,5,5′-tetrahydroxydiphenylmethane; (**5**) 15 2,2′,3-tribromo-3′,4,4′,5-tetrahydroxy-6′-hydroxymethyldiphenylmethane; (**6**) 6 and 3-bromo-4-(2,3-dibromo-4,5-dihydroxybenzyl)-5-methoxymethylpyrocatechol.

Bromophenols are marine secondary metabolites consisting of one or several benzene rings with variable levels of bromine and hydroxyl groups as substituents (see Figure 2). Bromophenols are produced predominantly by red macroalgae, although they have also been isolated from brown and green macroalgal species [93]. These compounds have shown promising health benefits, including anticancer, antioxidant and antidiabetic properties [93,94]. Recently, studies have also explored the use of bromophenols [61,62] and other halogenated molecules [63,64] isolated from multiple macroalgae for their antibacterial properties (see Table 1).

Oh et al. [61] isolated, characterized and induced modifications of several bromophenols from *Odonthalia corymbifera*. The compound 3,3′,5,5′-tetrabromo-2,2′,4,4′-tetrahydroxydiphenylmethane had strong antibacterial effects against *S. aureus*, *B. subtilis*, *M. luteus*, *P. vulgaris* and *S. Typhimurium*, while the synthetic bromophenols 3,3′-dibromo-6,6′-dihydroxydiphenylmethane and 3,3′,5,5′-tetrabromo-6,6′-dihydroxydiphenylmethane had also potent antibacterial effect against these bacteria, demonstrating the possibilities of inducing modifications in the naturally occurring bromophenols to generate novel halogenated compounds with antibacterial properties [61]. The antibacterial properties of bromophenols isolated from *Osmundaria colensoi* were also reported by Popplewell and Northcote [62].

Other studies had also confirmed and identified a wide variety of bromophenols with antibacterial properties from macroalgae [95,96], with promising applications as antifungal [97] and antiviral agents [98,99]. Further studies will be needed in order to clarify the antibacterial mechanisms of action of these promising compounds as well as the structure–function relationships of these molecules to be able to use them as natural antimicrobial preservatives for pharmaceuticals and food preservation purposes.

### 3.2. Proteins and Peptides

Previous reports have identified seaweeds as a promising source of protein with variable contents depending on the seaweed species and season of collection. In general, the protein content of seaweeds is described as low in brown seaweeds (3–15% DW), moderate in green algae (9–33% DW) and high for red seaweeds, reaching levels of up to 47% DW [100]. Proteins from macroalgae contain all the essential amino acids in higher amounts compared to terrestrial plants [101]. The mean values for individual amino acids were similar in brown, green and red algae, most species being particularly rich in glutamic and aspartic amino acids and deficient in methionine [102].

The relatively high amount of proteins in seaweed and their amino acid profile were key factors when considering macroalgal proteins as a source of bioactive peptides or cryptides. Bioactive peptides are short sequences of amino acids (2 to 30) that display powerful biological activities or health benefits when released for their parent proteins by enzymatic hydrolysis or other processes inducing mild protein degradation. The biological properties of bioactive peptides described to date include antihypertensive, antioxidant, antithrombotic, immuno-modulatory and antimicrobial properties [102]. Beaulieu et al. [65] extracted and hydrolyzed protein from *Saccharina longicruris* and identified that the hydrolysate fraction >10 kDa exhibited antibacterial properties against *S. aureus*. Following characterization and chemical synthesis of the peptides contained in the fraction, the authors did not find any antibacterial effect when using the peptides separately; however, their activity when mixed confirmed the previously mentioned antibacterial effects, establishing possible synergies between these compounds [65].

**Figure 3:**
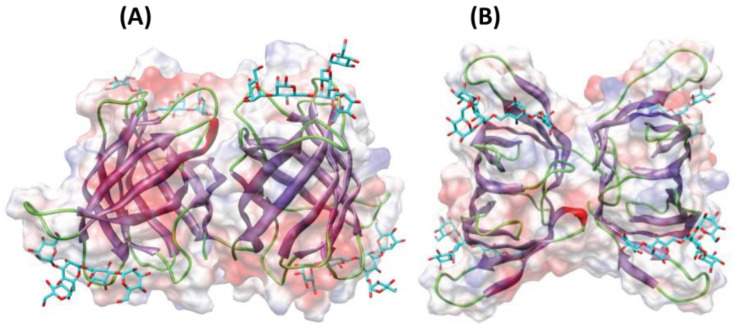
Ribbon diagrams showing the lateral (**A**) and front (**B**) views of the three-dimensional model built for lectins from macroalga *Agardhiella subulata* with a pentamannoside (colored in cyan). Surface electrostatic potential is shown in transparency and colored as red (for regions with negative electrostatic potential), blue (positive electrostatic potential) and grey (neutral). Image modified from Barre et al. [107].

Other relevant proteins with antimicrobial properties include lectins or agglutinins (see chemical structure in Figure 3). These compounds are glycoproteins found in a wide range of organisms with the ability to recognize and interact reversibly with free carbohydrates or glycoconjugates, without modifying their structure [103]. Due to this, lectins have been studied as antibiotic, anti-inflammatory, anti-adhesion, anticancer and antimicrobial agents [103]. To our knowledge, lectin from the algae *Microcystis viridis* is the only antimicrobial protein of algal origin currently included in the online database APD3 (The Antimicrobial Database) [104]. Moreover, lectins have recently gained attention as antiviral agents against coronavirus. O’Keefe et al. [105] reported high inhibition of SARS-CoV infection when using griffithsin (in vitro and in vivo) with very low toxicity, and promising results were also shown when using this compound against MERS-CoV [106].

### 3.3. Fatty Acids

**Figure 4:**
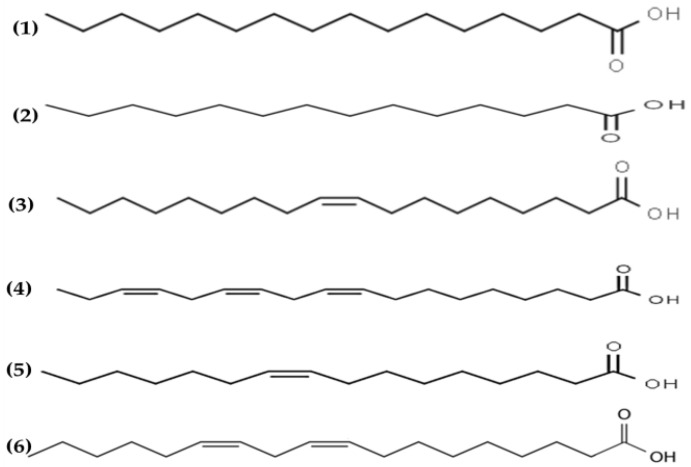
Chemical structures of (**1**) palmitic, (**2**) myristic, (**3**) oleic, (**4**) α linolenic, (**5**) palmitoleic and (**6**) linoleic acids isolated from macroalgae.

Antimicrobial lipids are defined as single-chain lipids or fatty acids and monoglycerides that interact with bacterial cell membranes and exhibit antibacterial activity [108]. Fatty acids are amphipathic molecules composed of a single saturated or unsaturated hydrocarbon chain (hydrophobic part) with a carboxylic acid group (hydrophilic part) on one end [108] (see Figure 4).

The antimicrobial potential of fatty acids was reported in the early 1960s by Katayama [109]. The characteristic fatty acid profile of seaweed usually contains saturated and unsaturated fatty acids, predominantly myristic, palmitic, oleic and eicosapentaenoic acids, which are associated with the antimicrobial properties of algae [110]. Fatty acids are ubiquitous molecules usually bonded to other components such as sugars, glycerol or phosphate groups to form lipids. The lipid content of seaweeds is usually low, ranging from 0.4 to 5% DW basis, but includes relevant lipids such as glycolipids and polyunsaturated fatty acids omega 3 (n3) with relevant biological properties and health benefits [111].

Several lipid extracts [66,67] and essential oils [68,69,70] obtained from macroalgae and their antimicrobial effects are summarized in Table 1. Park et al. [66] obtained lipid extracts from *Enteromorpha linza* containing mainly stearidonic and gamma-linolenic acids, with potent antimicrobial activities in vitro against *P. intermedia* and *P. gingivalis*. Lipid extracts from *Cladophora rupestris* containing mainly palmitic, myristic, oleic, α linolenic, palmitoleic and linoleic acids had powerful antibacterial activities [67]. Moreover, essential oils from multiple plants, consisting of a wide variety of compounds and secondary metabolites known to retard or inhibit the growth of bacteria, yeast and molds [2]. Recently, Patra, Das and Baek [69] obtained essential oils from *Laminaria japonica* containing 21 volatile compounds with antibacterial properties against foodborne pathogens. Similarly, essential oils from *Enteromorpha linza* and *Undaria pinnatifida* have been reported to have potent antimicrobial activities against several pathogens [68,70].

The mechanisms of antibacterial activity of fatty acids have been described in detail by Yoon et al. [108]. Overall, the amphipathic properties of fatty acids seem to play a major role in the membrane-lytic behavior of fatty acids that leads to membrane destabilization and pore formation and, thus, inhibition of cell growth (bacteriostatic action) or even cell death (bactericidal action). These compounds are also able to disrupt the electron transport chain (by binding to electron carriers or altering membrane integrity) and altering the process of oxidative phosphorylation (by decreasing the membrane potential and proton gradient), which are essential cellular processes for the production of energy. Moreover, fatty acids can directly inhibit membrane enzymes and also interfere in the nutrient uptake of the cells [108].

### 3.4. Polysaccharides

**Figure 5:**
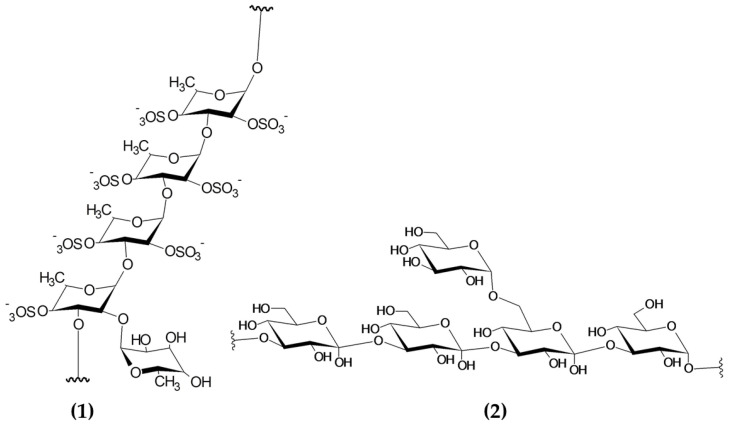
Chemical structure of building blocks of the polysaccharides (**1**) fucoidan (sulphated polysaccharide) and (**2**) laminarin (non-sulphated polysaccharide) from brown macroalgae. Image modified from Garcia-Vaquero et al. This Figure was reproduced from [55], with permission from Elsevier, 2021.

Seaweeds are some of the most valuable sources of polysaccharides, with variable contents normally ranging between 15 and 66% depending on the macroalgal species and other factors affecting the biomass (i.e., location, season) [112]. Polysaccharides are chemically described as compounds with a backbone of repeating monosaccharide units linked by glycosidic bonds. Macroalgal polysaccharides encompass a diverse group of molecules, including sulphated polysaccharides (i.e., fucoidan, ulvan and carrageenans) and non-sulphated polysaccharides (laminarin) [113]. The chemical structures of sulphated and non-sulphated polysaccharides from brown macroalgae are represented in Figure 5.

These compounds have recently attracted the attention of the scientific community due to their promising health benefits, including anticoagulant, antioxidant, antiviral, antitumor and immunostimulatory effects, in in vitro and/or in vivo model systems [55]. These health benefits are related to the chemical composition and structure of the macroalgal polysaccharides, being affected by the molecular weight, number and type of branches and sulphate content of the molecules, amongst other structural features [13,57,114].

Recent scientific literature summarized the promising antimicrobial effects of laminarin [73] and sulphated polysaccharides, mainly fucoidan extracted from brown macroalgae including *Sargassum* sp. [71,72,77], *Laminaria japonica* [74], *Spatoglossum asperum* [75] and *Fucus vesiculosus* [76]. However, these studies do not deal with pure and isolated individual molecules and, thus, the antimicrobial properties summarized in Table 1 refer mainly to the properties of extracts containing a variable mixture of chemical species including carbohydrates (concentrations ranging from 5 to 29%) and other minor compounds co-extracted from macroalgae.

The antimicrobial mechanisms of action of macroalgal polysaccharides have not yet been fully elucidated. Zhao et al. [115] suggested that the antibacterial capacity of fucoidan may be related to the contents of sulphuric acid and glucuronic acid released during the depolymerization of the molecules. According to these authors, these chemical species released from fucoidan have the capacity to bind to the bacterial membrane proteins, causing membrane cell disruption and cell death [115]. Gram-negative bacteria seem to be less sensitive to the antibacterial effects of fucoidan compared to Gram-positive. This fact was attributed to the presence of cell wall components that may act as a barrier for the antibacterial effects of fucoidan [115]. Palanisamy et al. [77] studied the antibacterial effects of fucoidan from *Sargassum polycystum* and appreciated a significant increase in the production of reactive oxygen species in all the microorganisms tested compared to the control. This fact suggests that fucoidan triggered the production of reactive oxygen species responsible for the cell membrane damage in the microorganisms [77]. 

Recent reviews also emphasize the use of non-sulphated polysaccharides such as laminarin [116]. However, very little is known about the biochemical pathways and interactions underlying the antimicrobial activities of laminarin and other non-sulphated polysaccharides. He et al. [117] studied the antimicrobial properties of polysaccharides produced by *S. virginia* against food spoilage and food poisoning microorganisms such as *S. aureus*, *B. subtilis*, *L. monocytogenes*, *E. coli*, *Zygosaccharomyces bailii* and *Candida utilis*. These authors suggested that the main targets of antibacterial polysaccharides may be the cell wall, cytoplasmic membranes and DNA [117].

### 3.5. Other Compounds

**Figure 6:**
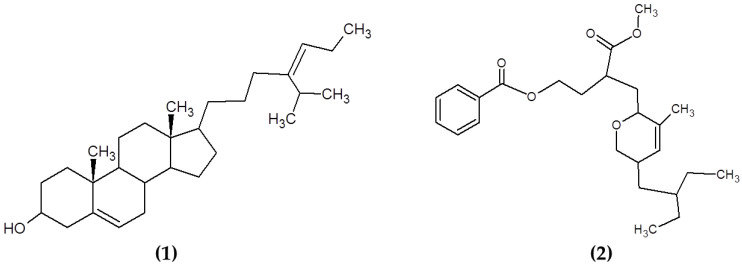
Chemical structure of (**1**) 24-propylidene cholest-5-en-3β-ol isolated from macroalgae *L. papillosa* and (**2**) the polyketide (3-(methoxycarbonyl)-4-(5-(2-ethylbutyl)-5,6-dihydro-3-methyl-2H-pyran-2-yl)-butyl benzoate) isolated from *S. myriocystum*.

Other compounds from macroalgae have also been studied for their antimicrobial properties, including sterols and polyketides. Sterols are a varied group of lipids from the steroid group present in the majority of eukaryotic cells, playing critical biological roles both as hormones and signaling molecules and in maintaining the membrane fluidity and permeability in eukaryotic cells. The structural features of these molecules include the presence of a fused four-ring core structure and a hydroxyl group (polar part of the molecule) at the 3-position of the A-ring. Sterols are highly diverse in nature and multiple routes of synthesis and differential chemical features can be appreciated between sterols from plants, fungi and animals [118]. Kavita, Singh and Jha [78] determined the antimicrobial properties of the sterol 24-propylidene cholest-5-en-3β-ol isolated from *Laurencia papillosa* against a wide variety of bacteria (see Figure 6). Similarly, previous reports on macroalgal sterols reported the powerful antimicrobial activities of these compounds [119,120,121]. The antimicrobial mechanisms of action of these compounds are still not fully elucidated, although previous studies suggested that the presence of sterols may influence the morphological responses in the cell membranes when triggered by antimicrobial lipids [122].

Polyketides are a diverse class of chemical compounds generated by enzymatic reactions that condense and modify acetate or propionate units through reduction, dehydration, cyclization and aromatization reactions [123]. Chakraborty et al. [79] isolated and characterized polyketides from the brown seaweed *Sargassum myriocystum* (Figure 6). They found that the compounds had a strong antibacterial activity against human opportunistic food pathogenic bacteria [79].

## 4. Incorporation of Antimicrobial Compounds from Macroalgae in Food Matrices

Despite the promising nutritional and health benefits of algae, limited products containing algae are currently available in the market. Whereas the human consumption of seaweeds has a long tradition and has enjoyed a huge reputation and continued consumption in Asian countries through millennia, the main use of seaweeds in western countries is mainly limited to the use of macroalgae or macroalgal-derived compounds as hydrocolloids, being incorporated into foods for their thickening and emulsifying properties [100,124]. Recent trends guided by an increased consumer preference towards “natural” and “sustainable” foods seem to create a promising scenario for the positioning of food products containing macroalgae in the market. In fact, the presence of seaweeds in stores and restaurants has increased in recent decades since the negative connotations previously attributed to seaweeds have vanished, being currently considered as a healthy, nutritious and tasty food [125].

Researchers have evaluated the use of seaweeds and algae as coloring agents or aiming to improve the nutritional and sensorial attributes of several food products. There are studies analyzing the incorporation of macroalgae or macroalgal ingredients into bakery and pasta products, meat and dairy products, mainly to improve the sensory attributes of foods and to enrich their mineral content or their health benefits (i.e., antioxidants) [12,14,126,127,128]. However, few studies are available evaluating the potential of macroalgae and/or macroalgal compounds against pathogenic and spoilage microorganisms in food, aiming to validate the antimicrobial functionality of macroalgae in real food matrices. Moreover, when incorporating macroalgae into real food products, one of the main factors limiting the use of these novel ingredients is the effect of these compounds on the sensory attributes of food, as this may influence their acceptability by consumers. The recent scientific literature evaluating the incorporation of these products as antimicrobial agents and the modifications appreciated in the sensory attributes of food (i.e., texture, odor and flavor) is summarized in Table 2.

### 4.1. Meat and Meat Products

The incorporation of macroalgae allows the lowering of the cholesterol, calories and fat added to these foods when designing new meat product formulations, while enriching them with the additional phytochemicals contained in macroalgae. Several authors studied the incorporation of small amounts of macroalgae (1–5% in the final product) into sausages [129,135] and frozen meat products such as beef patties [130] and restructured poultry steaks [131].

In general, the addition of small amounts of macroalgae increased the initial load of bacteria in the food products (see Table 2). This fact may be due to the initial bacterial load of the macroalgal samples as this may vary depending on the sampling site and also sampling procedures and pore-treatments applied to the biomass prior to their inclusion into food formulations. Recently, Barberi et al. [145] reported the presence of foodborne pathogens, such as *S. Typhimurium*, *V. parahaemolyticus* and enterohemorrhagic *E. coli* O157:H7, on kelp samples analyzed using molecular methods. The authors concluded that although the bacterial counts in the seaweeds were generally low, there is a risk for consumers associated with the presence of foodborne pathogens [145]. Thus, the pre-treatments of the macroalgal biomass, such as cleaning, drying and extraction procedures used to obtain macroalgal ingredients, may influence considerably the initial microbial loads of meat and other food products formulated using macroalgae and/or macroalgal ingredients.

Despite the inter-study differences in the microbial loads of meat products formulated using macroalgae, multiple studies confirmed the advantages of seaweeds when designing low-fat and low-salt meat products [129] and emphasized other advantages of the incorporation of seaweeds, such as reduced thawing and cooking losses [130,131] and modifications in the sensory attributes (i.e., texture, color, external appearance, aroma and flavor) that were considered acceptable when evaluating the products in sensory panels [130,131,135]. Cox and Abu-Ghannam [132] explored the incorporation of high amounts (10–40%) of *H. elongata* into frozen beef patties. Contrary to previous studies using low macroalgae incorporation, the authors appreciated low microbiological counts in patties containing macroalgae and no bacterial growth after day 30 of storage in patties containing ≥20% of macroalgae. Moreover, patties containing macroalgae had lower cooking losses and were tenderer compared to control patties without seaweed and were the preferred choice in terms of their sensory attributes according to the sensory panel, emphasizing improvements in texture and mouth feel [132].

To the best of our knowledge, limited studies are currently available on the incorporation of macroalgal extracts into meat. Moroney et al. [133] incorporated low levels of macroalgal extracts containing carbohydrates in minced pork patties (0.01–0.5%). The authors reported little or no effect in the microbial characteristic of the products when adding the seaweed extracts [133]. Similarly, Lorenzo et al. [134] found no antimicrobial effect on macroalgal extracts from *Ulva* spp. when added at 1000 mg extract per Kg in pork patties. The authors appreciated an increase in lactic acid bacteria and *Pseudomonas* during the storage of the products and significant effects of the macroalgal extract against the lipid oxidation of the patties [134].

### 4.2. Milk and Dairy Products

Few studies are currently available evaluating the effects of the addition of macroalgae or macroalgal extracts on the microbiological properties of milk and dairy products. The incorporation of low amounts of extracts (0.25 and 0.5%), containing phenols and other non-characterized molecules of diverse chemical nature, from *Ascophyllum nodosum* and *Fucus vesiculosus* was evaluated in milk [136] and yogurt [137]. The addition of macroalgal extracts to milk and yogurt had a significant influence on the color (yellowness and greenness) of the products and a positive influence on the lipid oxidation and shelf-life of the products, while no effects were found in the microbial counts between the seaweed-supplemented milk or yogurt compared to non-supplemented products [136,137].

Olmo, Picon and Nuñez [138] determined that the addition of 0.01% of five macroalgal species to cheese curd had no significant effect on cheese microbiota. Aerobic mesophilic and lactic acid bacteria counts varied by less than 0.3 log CFU/g, with low variation caused by the addition of seaweeds, and these cell counts declined during the ripening process by 1 log CFU/g in all cheeses [138]. In general, the counts of Gram-negative bacteria were under 4 log CFU/g and were below the detection level of the method from day 40 of ripening onwards. Other bacteria follow similar trends, including coliforms with counts under 3 log CFU/g and no-detectable counts from day 40 onwards. Yeasts and molds counts were non-detected on day 1 and increased by day 20 onwards, reaching levels of less than 2 log CFU/g for yeasts and 3 log CFU/g for molds, with no significant differences between cheeses [138]. Moreover, the supplementation with seaweeds influenced the antioxidant properties of cheese, increasing from day 20 onwards, particularly when adding *Himanthalia elongata*. Measurements of color and texture of cheese also varied widely when using seaweeds. Products supplemented with *H. elongata*, *U. pinnatifida* and *L. ochroleuca* received the highest odor and flavor quality scores by sensory panelists before day 60 of ripening, associated with low seaweed odor and flavor [138].

### 4.3. Other Food Products

Recent studies evaluating the microbiological quality of bakery products focused mainly on the effect of the addition of low amounts (0.1–2%) of macroalgal crude extracts containing a wide variety of compounds (see Table 2). In general, the addition of macroalgal extracts had a positive effect on reducing the cell counts of bread [139] and muffins [140], the products being acceptable when evaluated by sensory panels. Moreover, Kim et al. [141] found a 2 log decrease in the total viable cells and 3 log decrease in molds when supplementing bread with *S. sagamianum* extracts. The addition of these extracts altered significantly the color of the products and, thus, the incorporation of 0.25 and 0.5% of extract was preferred when evaluating the sensory attributes of bread [141].

The incorporation of extracts containing fucoidan suspensions (0.025–1%) into an apple beverage had a significant influence on the microbiological properties of the product [142]. Fucoidan concentrations in juice of 50 μg/mL had a bacteriostatic effect, while concentrations of 100–1000 μg/mL were bactericidal against *L. monocytogenes*. Moreover, the time required for the inactivation of *S. Typhimurium* decreased in a dose-dependent manner with the concentration of fucoidan applied in the product [142].

Other applications of macroalgal compounds also include the design of active coatings or edible films in contact with food to improve their microbiological properties. Neetoo, Ye and Chen [143] developed an edible coating containing alginate, κ-carrageenan, pectin, gelatin or starch to preserve cold smoked salmon slices and fillets. Edible coatings containing alginate were the most effective carrier for the various antimicrobial treatments in inhibiting the growth of *L. monocytogenes*. Moreover, when the fillets and slices were inoculated with 500 CFU/cm^2^ and stored at 4 °C for 30 days, supplemented alginate coatings significantly delayed the growth of *L. monocytogenes* during storage, with final counts reaching 4.1–3.3 log CFU/g in slices or 4.4–3.8 log CFU/g in fillets, while the untreated salmon reached counts of 7.3 and 6.8 log CFU/g for slices and fillets, respectively [143]. Moreover, seaweed extracts from *H. elongata* and *P. palmata* were effective in formulating edible films with high antioxidant activity that were useful in controlling pH and water activity changes during the storage of fish burgers and reduced microbial growth, especially when using *H. elongata* [144].

## 5. Future Trends and Challenges of the Use of Antimicrobials from Seaweeds for Food Applications

As seen in the recent literature, macroalgae and macroalgal compounds offer almost an unlimited field of study and potential for bio-mining multiple compounds with antimicrobial potential due to the extreme variability of both the biomass and the chemical species produced by seaweeds influenced by environmental stressors affecting their survival and growth. Multiple compounds have been isolated and characterized from macroalgae to date and novel molecules are continually being discovered, including phenols (phlorotannins, bromophenols and other halogenated compounds), fatty acids, carbohydrates, proteins, peptides and other minor compounds, such as sterols and polyketides, with a wide range of antimicrobial potential and huge possibilities for food and non-food applications [51,52,53,54].

Despite the promising molecules produced by macroalgae and their potential applications, the incorporation of full macroalgal biomass into food products can also represent a safety hazard. Macroalgae can be the source of chemical hazards, mainly metals; less frequently, other biological hazards, due to the contamination of the biomass with *Salmonella* or biotoxins produced by dinoflagellates; or physical hazards, such as micro- and nano-plastics [146,147].

As metals are commonly described as safety hazards in macroalgae, the levels of certain metals are regulated in the European Union (EU) for macroalgae when used for food and feed applications by the Regulation (EC) No. 1881/2006 [148] and the Directive 2002/32/EC [149], respectively. The maximum levels of metals in macroalgae when used as feed (in mg per kg of feed with a moisture content of 12%) were established for arsenic (As, 40 mg total As/kg or 2 mg inorganic As/kg), cadmium (Cd, 1 mg/kg), lead (Pb, 10 mg/kg) and mercury (Hg, 0.1 mg/kg) [149]. Currently, there are no maximum levels established for any metal for macroalgae when used as food [148]. In the case of food supplements, the maximum allowed levels of metals in macroalgae are 3 mg/kg wet weight (ww) for Cd and Pb and 0.10 mg/kg ww for Hg [148].

In 2018, the EU published specific recommendations for the monitoring of As (total and inorganic forms), Cd, Pb, Hg (total and organic forms) and iodine (I) in macroalgae used for both food and feed applications [150]. Thus, it is expected that new threshold or maximum levels of these metals in macroalgae for food applications will be established in the near future. Despite the presence of these metals, further information is needed on how processing affects the concentration and bioavailability of these contaminants. Previous studies reported a decrease of up to 60% of the total As in macroalgae when washing and soaking the biomass before cooking [151]. Moreover, Ichikawa et al. [152] observed an accumulation in mice of only 5% of the total As of washed and cooked *Hizikia fusiforme* after the digestion process.

Thus, the use of extracts in food products seems to be a more promising strategy to explore in the future to decrease or reduce the safety hazards of the use of full macroalgal biomass in foods. The majority of the recent scientific literature on novel antimicrobials from macroalgae focused on determining the antimicrobial potential in vitro of undetermined mixtures of multiple and non-characterized chemical species extracted from seaweeds using a wide variety of solvents [57]. The isolation and chemical characterization of individual compounds will help to clarify and elucidate the antimicrobial mechanisms of action and the structure–function relationships of these compounds as most of these mechanisms remain relatively unknown.

Moreover, although naturally occurring marine products have distinctive chemical and stereochemical structural features [11], the synthesis and modification of these compounds by means of synthetic chemistry may be useful when tailoring natural products and modify or increase their efficiency against pathogens by obtaining new chemical entities [153]. The chemical synthesis of the most active compounds identified from macroalgae could also help to scale-up the exploitation of these molecules at an industrial level by producing more uniform chemical compounds, compared to the variable structure of the molecules appreciated when extracting them from natural sources, and without compromising the future supply of macroalgae and the sustainability of the marine environment when using wild harvested biomass to produce these compounds. Furthermore, the establishment and exploitation of macroalgal farms could also represent a promising scenario for the sustainable exploitation of these molecules. Recent advances in sequencing and “-omics” technologies have been applied to discover natural compounds [11]. Genomic sequencing and metagenomics are currently driving a new era of genome-guided investigations that could complement the traditional bioactivity-guided methods by tailoring the search for novel compounds to the most promising molecules. These strategies offer an invaluable insight into the biosynthetic pathways of antimicrobials and the evolutionary and defensive strategies of these organisms in the marine environment [11].

## 6. Conclusions

There is a significant amount of scientific literature on the antimicrobial effects in vitro of macroalgae or non-purified and/or characterized macroalgal extracts consisting of a mixture of compounds of variable chemical nature. Further studies are needed concerning the extraction, chemical characterization and incorporation of these promising compounds against foodborne pathogens and other food spoilage bacteria into real food matrices to develop this market further and to increase the potential of natural antimicrobials in food. The efficiency of these compounds against several food pathogens must also include an evaluation of the practical doses that can be used of these natural products in food without affecting negatively the sensory attributes of these products, as this may negatively influence the consumption of food containing natural products from macroalgae. Moreover, it is necessary to explore further the chemical structure of seaweed antimicrobials and the structure–function relationship of these molecules in order to understand their influence on the storage conditions and shelf-life, quality and health attributes of novel, fortified food products containing them.

## Figures and Tables

**Table 1:** Summary of the main compounds isolated and characterized from macroalgae with antimicrobial effects against several pathogens when assayed in vitro in the scientific literature from 2006 to 2020.

Chemical Compounds	Source	Activity Against Bacterial Pathogens	References
**Phlorotannins**			
Dieckol	*Ecklonia stolonifera*	Methicillin-resistant *Staphylcoccus aureus* (MRSA) (MIC 64 μg/mL) and methicillin-susceptible *S. aureus* (MSSA) (MIC 32 μg/mL).	Lee et al. [58]
Eckol	*Ecklonia cava*	Inhibition of 17 strains of *S. aureus* MRSA (MIC 125-250 μg/mL) and 16 strains of *Salmonella* spp. (MIC 250 μg/mL).	Choi et al. [59]
Fucofuroeckol-A	*Eisenia bicyclis*	*Propionibacterium acnes* (KCTC 3314) (MIC 32 µg/mL), *P. acnes* isolate 2875 (MIC 32 µg/mL), *P. acnes* isolate 2876 (MIC 64 µg/mL), *S aureus* (KCTC 1927) (MIC 128 µg/mL) and *Staphylococcus epidermidis* (KCTC 1370) (MIC 64 µg/mL).	Lee et al. [60]
**Bromophenols**			
2,20,3,30-tetrabromo-4,40,5,50- tetrahydroxydiphenylmethane	*Odonthalia corymbifera*	*S. aureus* ATCC6538p (MIC 25 μg/mL), *Bacillus subtilis* ATCC 6633 (MIC 25 μg/mL), *Micrococcus luteus* IFC 12708 (MIC 25 μg/mL), *Proteus vulgaris* ATCC3851 (MIC 50 μg/mL), *S. Typhimurium* ATCC 14028 (MIC 50 μg/mL) and *E. coli* ATCC 25922 (MIC > 100 μg/mL).	Oh et al. [61]
2,20,3-tribromo-30,4,40,5-tetrahydroxy-60 -hydroxymethyl diphenylmethane	*O. corymbifera*	*S. aureus* ATCC6538p (MIC 50 μg/mL), *B. subtilis* ATCC 6633 (MIC 100 μg/mL), *M. luteus* IFC 12708 (MIC 50 μg/mL), *P. vulgaris* ATCC3851 (MIC 100 μg/mL), *S. Typhimurium* ATCC 14028 (MIC 100 μg/mL) and *E. coli* ATCC 25922 (MIC > 100 μg/mL).	Oh et al. [61]
3-bromo-4-(2,3-dibromo-4,5-dihydroxybenzyl)-5- methoxymethylpyrocatechol	*O. corymbifera*	*S. aureus* ATCC6538p (MIC 25 μg/mL), *B. subtilis* ATCC 6633 (MIC 25 μg/mL), *M. luteus* IFC 12708 (MIC 25 μg/mL), *P. vulgaris* ATCC3851 (MIC 25 μg/mL), *S. Typhimurium ATCC* 14028 (MIC 25 μg/mL) and *E. coli* ATCC 25922 (MIC > 100 μg/mL).	Oh et al. [61]
Lanosol methyl ether	*Osmundaria colensoi*	*Mycobacterium smegmatis* (IC_50_ 7.8 μM).	Popplewell and Northcote [62]
Lanosol butanone	*O. colensoi*	*M. smegmatis* (IC50 26.2 μM).	Popplewell and Northcote [62]
Rhodomelol	*O. colensoi*	*M. smegmatis* (IC50 28.1 μM).	Popplewell and Northcote [62]
**Other halogenated compounds**			
Bromoform and dibromoacetic acid	*Asparagopsis armata*	Both methanol and dichloromethane extracts from *A. armata* were active against 6 strains of bacteria (*Vibrio harveyii*, *Vibrio alginolyticus*, *Pseudomonas aeruginosa*, *S. aureus*, *S. epidermis* and *E. coli*). In general, *E. coli* and *P. aeruginosa* were most susceptible to the dichloromethane extracts.	Paul et al. [63]
Sphaerodactylomelol	*Sphaerococcus coronopifolius*	*S. aureus* (MIC 96 µM).	Rodrigues et al. [64]
Bromosphaerol	*S. coronopifolius*	*S. aureus* (MIC 22 µM).	Rodrigues et al. [64]
12R-hydroxybromosphaerol	*S. coronopifolius*	*S. aureus* (MIC 6 µM).	Rodrigues et al. [64]
**Proteins and peptides**			
Protein hydrolysate >10 kDa containing 9 peptides (TITLDVEPSDTIDGVK, ISGLIYEETR, MALSSLPR, ILVLQSNQIR, ISAILPSR, IGNGGELPR, LPDAALNR, EAESSLTGGNGCAK and QVHPDTGISK)	*Saccharina longicruris*	Decrease the maximum specific growth rate of *S. aureus* at concentrations ranging from 0.31 to 2.5 mg/mL.	Beaulieu et al. [65]
**Fatty acids**			
Stearidonic and gamma-linolenic acids	*Enteromorpha linza*	*Prevotella intermedia* (MIC 39.06 μg/mL) and *Porphyromonas gingivalis* (MIC 9.76 μg/mL). Both compounds were also active against *Aggregatibacter actinomycetemcomitans*, *Candida albicans*, *Fusobacterium nucleatum* subsp. *vincentii* and *Streptococcus mutans*.	Park et al. [66]
Lipid extract containing palmitic, myristic, oleic, α linolenic, palmitoleic and linoleic acids	*Cladophora rupestris*	Antibacterial activity against *Enterococcus* sp., *Streptococcus agalactiae* and *Vibrio cholera* non-O1.	Stabili et al. [67]
Essential oil composed of acids (54.6%), alkenes (21.1%), alcohols (4.5%), aldehydes (3.7%) and ketones (2.8%)	*E. linza*	*E. coli* ATCC 43889 and 43890 (MIC 12.5 mg/mL; MBC 25 mg/mL), *S. Typhimurium* ATCC 19586 (MIC 25 mg/mL; MBC 25 mg/mL) and *S. Typhimurium* ATCC 43174 (MIC 12.5 mg/mL; MBC 25 mg/mL).	Patra et al. [68]
Essential oil composed of tetradeconoic acid (51.75%), hexadecanoic acid (16.57%), (9Z,12Z)-9,12-Octadecadienoic acid (12.09%) and (9Z)-hexadec-9-enoic acid (9.25%)	*Laminaria japonica*	*S. aureus* (11.5 ± 0.58 mm inhibition zone at 25 mg/disc) and *Bacillus cereus* (10.5 ± 0.57 mm inhibition zone at 25 mg/disc), but no inhibition of *E. coli* O157:H7.	Patra et al. [69]
Essential oil composed of tetradecanoic acid (31.32%) and hexadecanoic acid (22.39%) was present in the highest amount, followed by 3-hexen-1-ol (5.67%), erythritol (4.73%), 4-imidazolidinone (4.40%) and (9Z)-hexadec-9-enoic acid (4.37%)	*Undaria Pinnatifida*	*S. aureus* ATCC 12600 (MIC 12.5 mg/mL; MBC 25 mg/mL) and *S. Typhimurium* ATCC 43174 (MIC 25 mg/mL; MBC 25 mg/mL).	Patra et al. [70]
**Polysaccharides**			
Crude extracts containing fucoidan (sulphate content: 5.3 ± 1.54%)	*Sargassum swartzii*	10 µg extract inhibited: *S. aureus* (9 ± 0.67 mm inhibition), *Proteus vulgaris* (7 ± 0.72 mm inhibition), *E. coli* (15 ± 0.28 mm inhibition), *B. subtilis* (16 ± 0.52 mm inhibition), *P. aeruginosa* (11 ± 0.48 mm inhibition), *Salmonella* Typhi (10 ± 0.62 mm inhibition), *Shigella flexineri* (6 ± 0.78 mm inhibition), *Enterococcus faecalis* (10 ± 0.68 mm inhibition) and *Aeromonas hydrophilla* (2 ± 0.32 mm inhibition).	Vijayabaskar et al. [71]
Fucoidan (sulphate content: 29.26 ± 0.83%)	*Sargassum wightii*	*P. aeruginosa* (MIC 62.5 μg/mL; MBC 150 μg/mL) and *E. coli* (MIC 125 μg/mL; MBC 250 μg/mL).	Marudhupandi and Kumar [72]
Laminarin-rich extracts (no purity reported)	*Laminaria hyperborea* and *Ascophyllum nodosum*	*E. coli* (MIC non detected (nd)-596.8 mg/mL), *S. Typhimurium* (13.1–33.4 mg/mL) *S. aureus* (nd-66.8 mg/mL) and *L. monocytogenes* (nd-66.8 mg/mL).	Kadam et al. [73]
Fucoidan	*L. japonica*	Unprocessed fucoidans did not show obvious antibacterial activity against *E. coli* and *S. aureus* even at 10 mg/mL. Depolymerized fucoidans effectively inhibit the proliferation of both bacteria.	Liu et al. [74]
Fucoidan (21.35 ± 0.81% sulphate)	*Spatoglossum asperum*	*A. hydrophila* (MIC 100 μg/mL).	Palanisamy et al. [75]
Fucoidan (sulphate content: 14 ± 2.7%)	*Fucus vesiculosus*	*L. monocytogenes* KCTC 13064 (MIC 250 µg/mL), *S. aureus* KCTC 3881 (MIC 500 µg/mL), *E. faecalis* KCTC 5289 (MIC 1000 µg/mL), *S. mutans* KCTC 5458 (MIC 125 µg/mL), *S. mutans* KCCM 40105 (MIC 250 µg/mL), *Streptococcus oralis* KCCM 41567 (MIC 500 µg/mL), *Streptococcus sobrinus* KCTC 5809 (MIC 250 µg/mL), *S. sobrinus* KCCM 11898 (MIC 250 µg/mL), *Streptococcus sanguinis* KCTC 5643 (MIC 500 µg/mL), *Lactobacillus acidophilus* KCTC 3164 (MIC 500 µg/mL) and *Streptococcus thermophilus* KCTC 3658 (MIC 500 µg/mL).	Jun et al. [76]
Fucoidan (sulphate content: 20.41 ± 0.91%)	*Sargassum polycystum*	*S. mutans* (MIC 100 μg/mL; MBC 300 μg/mL), *P. aeruginosa* (MIC 50 μg/mL; MBC 200 μg/mL), *S. aureus* (MIC 200 μg/mL; MBC 300 μg/mL) and *E. coli* (MIC 200 μg/mL; MBC 300 μg/mL).	Palanisamy et al. [77]
**Other compounds**			
Sterol (24-propylidene cholest-5-en-3β-ol)	*Laurencia papillosa*	Antibacterial activity against *E. coli*, *P. aerugenosa*, *Klebsiella pneumonia* and *S. flexineri*. MIC ranging from 1.2 to 1.7 μg/mL (IC_50_).	Kavita et al. [78]
Polyketide (3-(methoxycarbonyl)-4-(5- (2-ethylbutyl)-5,6-dihydro-3-methyl2H-pyran-2-yl)-butyl benzoate)	*Sargassum myriocystum*	*Vibrio parahemolyticus* (zone of inhibition 7 mm at 10 µg/disk), *Vibrio vulnificus* (zone of inhibition 7 mm at 10 µg/disk) and *A. hydrophilla* (zone of inhibition 8 mm at 10 µg/disk).	Chakraborty et al. [79]
Polyketide (2-(8-butyl-3-ethyl-3,4,4a,5,6, 8ahexahydro-2H-chromen-6-yl)-ethyl benzoate)	*S. myriocystum*	*V. parahemolyticus* (zone of inhibition 9 mm at 10 µg/disk), *V. vulnificus* (zone of inhibition 8 mm at 10 µg/disk) and *A. hydrophilla* (zone of inhibition 7 mm at 10 µg/disk).	Chakraborty et al. [79]

**Table 2:** Summary of the incorporation of macroalgae or macroalgal extracts into real food matrices and the effects of these compounds on the antimicrobial and sensory attributes of foods.

Food Product	Macroalgae or Etract	Microorganisms Tested	Antimicrobial Effect	Effects on the Quality Attributes of Food	References
**Meat products**					
Sausages (frankfurter)	*Himanthalia elongata* dried. Addition to food: 5%.	Total viable count, lactic acid bacteria and *Enterobacteriaceae* counts.	Sausages containing seaweed had high total viable count from day 14 of storage, with lactic acid bacteria becoming the predominant microflora. *Enterobacteriaceae* count was always below 2 log CFU/g.	Seaweed allowed the development of low-fat frankfurters with favorable sensory properties (hard and chewy) with good water and fat binding properties compared to non-supplemented sausages. Macroalgae are useful in the production of products with reduced salt contents.	López-López et al. [129]
Frozen beef patties	*Undaria pinnatifida* dried. Addition to food: 3%.	Viable aerobic microorganism and *Enterobacteriaceae* counts.	Microbial populations generally increased with the addition of macroalgae, although no significant differences were appreciated in microbial populations with respect to control patties over time. Total viable counts were in the range of 6–6.4 log CFU/g and *Enterobacteriaceae* did not exceed 4.3 log CFU/g.	Patties with macroalgae had less thawing and cooking losses and were softer compared to control patties, while having a higher mineral content. All the products were considered acceptable by a sensory panel. The addition of macroalgae did not affect the properties of the patties in the course of frozen storage.	López-López et al. [130]
Frozen restructured poultry steak	*H. elongata* dried. Addition to food: 3%.	Total viable count, lactic acid bacteria, *Enterobacteriaceae*, β-glucuronidase-positive *E. coli* and *Salmonella* spp.	Products with macroalgae had higher levels of total viable counts and lactic acid bacteria compared to control.	The incorporation of seaweed caused an increase in purge loss and a reduced cooking loss. All the steaks were judged acceptable by a sensory panel.	Cofrades et al. [131]
Frozen beef patties	*H. elongata* powder. Addition to food: 10–40%.	Total viable counts.	Microbiological counts were low in patties containing macroalgae. No bacterial growth was appreciated after day 30 of storage in patties containing ≥20% of macroalgae.	Patties with seaweed showed reduced cooking losses and were nearly 50% more tender as compared to patties without seaweed. The lipid oxidation, dietary fiber and antioxidant properties of patties were improved by the addition of macroalgae. Sensory analysis (aroma, appearance, texture and taste) indicated that the seaweed patties were accepted by consumers, the patties containing 40% seaweed being preferred overall due to improvements in texture and mouth feel.	Cox and Abu-Ghannam [132]
Minced pork patties (fresh and cooked)	Macroalgal extracts (laminarin (9.3%) and fucoidan (7.8%)). Addition to food: 0.01–0.5%.	Total viable counts.	No effect on the microbial population studied.	The addition of macroalgal extracts had no effect on pH, water holding capacity and cook loss of patties. The patties containing 0.01% of macroalgal extract were preferred by the sensory panelists.	Moroney et al. [133]
Pork patties	*Ulva* spp. extract (acidic extract). Addition to food: 1000 mg extract per kg.	Total viable counts, lactic acid bacteria and *Pseudomonas*.	Macroalgal extracts did not affect the microbial growth in pork patties compared to control. Lactic acid bacteria and *Pseudomonas* increased during storage in patties with macroalgae.	The addition of macroalgal extract was effective against lipid oxidation, although not enough to maintain color stability during the storage of the patties.	Lorenzo et al. [134]
Sausages (frankfurters)	*Porphyra umbilicalis*, *Palmaria palmata*, *H. elongata* and *U. pinnatifida*. Addition to food: 1%.	Total viable counts.	Sausages containing macroalgae had higher total viable counts at day 15 compared to control with bacterial counts exceeded by day 55 of storage. The frankfurters containing *U. pinnatifida* had higher counts compared to the other macroalgae used.	Significant differences in sensory attributes (color, external appearance, aroma, flavor and texture) were different depending on the macroalgae used. Reformulated frankfurters containing *H. elongata* were better accepted compared to the other macroalgae.	Vilar et al. [135]
**Milk and dairy products**					
Raw whole cow’s milk	*Ascophyllum nodosum* and *Fucus vesiculosus* extracts (aqueous and methanolic extracts). Addition to food products: 0.25 and 0.5%.	Total plate count, total coliforms, yeasts and molds.	No effect on the studied microorganisms.	Macroalgal extracts were stable in milk and their antioxidant activities were high before and after an in vitro digestion. Macroalgal extracts improved milk quality and shelf life characteristics.	O’Sullivan et al. [136]
Yogurt	*A. nodosum* and *F. vesiculosus* extracts (aqueous and methanolic extracts). Addition to food: 0.25 and 0.5%.	*S. thermophilus* and *Lactobacillus delbrueckii* subsp. *bulgaricus*.	No effect on the studied microorganisms.	No effect on pH, whey separation or negative effects on shelf-life of yogurt. Increased yellowness and reduced levels of lipid oxidation in product containing macroalgal extracts. The addition of macroalgal extracts had no effect on the antioxidant properties of the yogurt. Yogurts containing *A. nodosum* aqueous extract were preferred by the sensory panelists.	O’Sullivan et al. [137]
Cheese	Dried macroalgae (*H. elongata*, *Laminaria ochroleuca*, *P. umbilicalis*, *Ulva lactuca* and *U. pinnatifida*). Addition to food: 0.1%.	Lactic acid bacteria, lactobacilli, enterococci, Gram-negative bacteria, coliforms, yeasts and molds.	Levels of all bacteria were similar in all cheeses. The levels of Gram-negative and coliforms were below the limit of quantification from day 40 of ripening onwards in cheeses containing *P. umbilicalis* and *U. lactuca*.	Cheese with macroalgae had increased whey retention and moisture content and a lower pH compared to control. The supplementation with *H. elongata* was preferred to the other macroalgae analyzed due to the improved sensory and antioxidant characteristics of cheese.	del Olmo et al. [138]
**Bakery products**					
Bread	*Myagropsis myagroides* ethanolic extracts. Addition to food products: 0.5, 1 and 2%.	Total viable counts.	Decreased total microbial count was appreciated in bread containing 2% of macroalgal extract.	Breads containing 0.5% of extract had improved shelf-life, overall quality and acceptance compared to non-supplemented bread.	Lee et al. [139]
Muffins	*Ecklonia cava* hot water extracts. Addition to food products: 0.1, 0.5 and 1%.	Total viable counts.	Muffins containing 1% of extract had a low total microbial count during storage compared to control products.	All supplemented muffins had acceptable sensory attributes (color, flavor, taste, texture and overall acceptability). The antioxidant activities of the products increased with the addition of macroalgal extracts in the products.	Jung et al. [140]
Bread	*Sargassum sagamianum* extract. Addition to food: 0.25, 0.5 and 0.75%.	Total viable counts and mold.	Bread containing extracts had reduced total microbial counts (2 log cycles) and mold counts (3 log cycles) compared to non-supplemented bread.	No changes in moisture content or pH (days 3 to 9). Yellowness of the product increased due to the addition of the macroalgal extract. Breads containing 0.25 and 0.5% of extract had preferable sensory attributes (taste, total preference, inner shape and color) compared to control and highly supplemented breads.	Kim et al. [141]
**Beverages**					
Ultra-high temperature processed apple beverage	*F. vesiculosus* (fucoidan extract ≥95% purity). Addition to food: 25, 100 and 1000 μg/mL.	*L. monocytogenes* and *S. Typhimurium* (CECT 443).	No effect of fucoidan was detected on *L. monocytogenes* in beverages at concentrations of 5–25 μg/mL. When the concentration of fucoidan increased to 50 μg/mL, a bacteriostatic effect was appreciated and concentrations of fucoidan of 100–1000 μg/mL were bactericidal. The higher the concentration of fucoidan added, the shorter the exposure time required to completely inactivate *S. Typhimurium* in the food matrix. In addition, 1000 μg/mL fucoidan reduced the *S. Typhimurium* initial counts by 4 log cycles after 5 days of exposure incubated at 8 °C.	The organoleptic properties of the apple juice remained intact after the addition of fucoidan (25–1000 μg/mL).	Poveda-Castillo et al. [142]
**Fish and seafood products**					
Cold smoked salmon slices and fillets	Alginate and carrageenan edible films.	*L. monocytogenes* (PSU1, PSU9, F5069, ATCC 19115 and Scott A), anaerobic and aerobic bacteria counts.	Alginate coating was the most effective film at inhibiting the growth of *L. monocytogenes*. Coatings also suppressed the growth of spoilage aerobes and anaerobes, with populations 3.7–4.0 and 2.8–3.0 log CFU/g lower than untreated controls after 30 days of refrigeration.	-	Neetoo et al. [143]
Fresh fish burgers	*H. elongata* and *P. palmata* edible films containing macroalgae or macroalgal aqueous extracts.	Total aerobic mesophilic and total psychrotrophic bacteria.	Edible films with *P. palmata* and macroalgal extracts were less effective in reducing the microbial growth of total aerobic mesophilic and total aerobic psychrotrophic bacteria compared to films containing macroalga *H. elongata*.	Edible films with macroalgae controlled effectively the pH and water activity changes over storage of fish burgers. Reduced lipid oxidation and increased antioxidant capacity of trout burgers over storage when using edible films with macroalgae.	Albertos et al. [144]

## Data Availability

Not applicable.
